# Development process and user experiences of an online support program for first-time hearing aid users

**DOI:** 10.3389/fdgth.2026.1924050

**Published:** 2026-10-14

**Authors:** Sandra Ahlberg, Linda Ivarsson, K. Jonas Brännström, Marie Öberg, Elisabet Sundewall Thorén

**Affiliations:** 1 Logopedics, Phoniatrics and Audiology, Department of Clinical Sciences, Lund University, Lund, Sweden; 2 Department of Otorhinolaryngology, Head and Neck Surgery, Audiology Clinic, Skåne University Hospital, Lund, Sweden; 3 Department of Otorhinolaryngology in Östergötland, Linköping University Hospital, Linköping, Sweden; 4 Department of Biomedical and Clinical Sciences, Linköping University, Linköping, Sweden

**Keywords:** aural rehabilitation, eHealth, hearing aid users, support program, teleaudiology

## Abstract

**Purpose:**

Many with hearing loss do not recognize their need for support or may struggle to acknowledge and accept their hearing difficulties, and when one receives hearing aids for the first time, many feel the need for more information and support during their rehabilitation process. The present article describes the development process of an online program in Swedish that supports first-time hearing aid users in an interleaved manner with the traditional audiological rehabilitation at the clinic.

**Method:**

In this formative evaluation study, the outline and content of the program was inspired by existing literature and informed by clinical expertise. The program was implemented within the Swedish national health portal. At the end of an iterative development process, four first-time and seven experienced hearing aid users evaluated the program in parallel to the provision of their new hearing aids and provided structured feedback on comprehension, relevance, manageability and ease of use to inform further development.

**Outcomes:**

Participant feedback identified the need for some minor changes but that the program was considered useful to first-time hearing aid users, comprehensible, relevant and manageable.

**Conclusions:**

The evaluation from the end-users demonstrated that all parts of the online support program were relevant for first-time hearing aid users, and that it was manageable and easy to use. However, content regarding assistive listening devices was deemed less relevant and some participants experienced difficulties with logging in to the national health portal.

## 1 Introduction

Individuals going through audiological rehabilitation receive tools for sensory management, instructions to improve outcomes of sensory management, perceptual training through everyday activities and counselling to improve participation and quality of life (1).

Much effort during audiological rehabilitation is laid on sensory management including fitting of hearing aids and instruction on how to use these (1). Despite this focus, many individuals are reported to discontinue using their hearing aids (2). Two of the most common reasons are hearing aid management difficulties and lack of perceived benefit (3, 4). Earlier studies have shown that many with hearing problems request more information and support during audiological rehabilitation, which could be a way to increase the use of hearing aids (5, 6). However, since a large amount of information is provided in a limited amount of clinical time, previous studies found that some information is simply forgotten (7–10). This is in line with other studies that suggest that patients forget between 40% and 80% of medical information if it is provided verbally (11). Perceptual training aims to target speech perception in daily life and can be practiced through engagement in everyday activities or specific training programs (1). Further, counselling involving emotional support is often overlooked and research has suggested that additional support within this domain could strengthen the rehabilitation process through a therapeutic relationship, shared decision making and involvement in one's audiological rehabilitation (12).

Thus, to support the audiological rehabilitation process, additional support with high availability can be added, along with counselling and instruction for self-management skills aiming to reduce residual activity limitations and participation restrictions (13–15). However, since it is common to forget or misremember verbal information at medical visits, the use of complementary visual and written support to understand and remember this information correctly are recommended (11).

This additional support could, for example, be added alongside standard clinical practice. The standard clinical practice in Sweden often consists of 3-4 clinical visits with an audiologist. Briefly described, these visits can be explained as follows: during the first visit an audiological assessment including clinical tests and the individual's hearing situation is conducted. Further, a choice of hearing aids to try is made and rehabilitation goals are discussed. At the second visit the hearing aids are fitted and instructions on how to handle and manage these are provided. At the third visit focus lays on fine tuning and repetition on handling and management of the hearing aids and communication strategies are often discussed. At the fourth visit, which is often the final, further fine tuning and repetition of information is provided. After these visits, the individual is responsible to contact the audiologist if further adjustments or support is needed.

To support people with hearing problems beyond hearing aid fitting several audiological support programs have been developed which have demonstrated positive effects (14, 16–21). Some are delivered as group rehabilitation, and some are individualized (22, 23). They can be given in person, online, or as a hybrid intervention, and are often designed to complement or serve as an alternative to hearing aid fitting (24–26). Examples of audiological rehabilitation programs include those of Thorén, Öberg (16), who created and tested an online intervention for adult hearing aid users, and Ratanjee-Vanmali, Swanepoel (27), who investigated a hybrid hearing health service. Furthermore, C2Hear and m2Hear are two multimedia educational programs designed to complement the clinical rehabilitation practices (28, 29). Also, Meijerink, Pronk (19) assessed the effectiveness of an e-mail-based support program given as an addition to hearing aid fitting for both first-time and experienced hearing aid users. However, to our knowledge, there is a lack of an online program for audiological rehabilitation that supports and follows the individual in a structured way from the decision to get hearing aids until the final follow-up.

Based on these previous findings, the present study describes the development of an online program that aims to provide information, support and guidance in parallel with standard clinical practice.

## 2 From idea to initial version

This study was conducted under the approval of the Swedish Ethical Review Authority (DNR 2022-01982). Informed written consent was gathered from all participants.

### 2.1 Initial considerations, functions and online portal selection

Several requirements for the present program were identified. One requirement was that the program could be provided to individuals receiving audiological rehabilitation parallel with their clinical visits. The intention was to support clinical activities with patient preparations before visits and remind individuals of both information and the rehabilitation process outside the clinical visits. A second requirement was that it would be possible for the individual to work with the program wherever and whenever. A third requirement was the inclusion of a chat function, enabling users to ask their audiologist questions and receive personalized feedback when needed. Lastly, the program was required to be easily implemented into the clinical workflow.

Based on these requirements, the Swedish national health portal called ‘1177.se’ was selected to host the online support program. In 2023, 81% of the adults in Sweden had used 1177.se (30). 1177.se is a secure portal that gives citizens online access to information about regional healthcare and illnesses, symptoms, and treatments along with access to their electronic health records. Additionally, they can get access to a section of the portal called the ’Support and Treatment’ platform. The ’Support and Treatment’ platform allows for the creation and assignment of support and treatment programs and have been used in recent hearing-related studies (31, 32).

### 2.2 Content development

The content of the program was inspired by existing literature on audiological rehabilitation (16, 19, 21, 33–35) and by clinical, audiological expertise. Boothroyd (1) suggested that audiological rehabilitation is based on four components: sensory management, instruction to improve outcomes of sensory management, perceptual training through everyday activities and counselling to improve participation and quality of life. The content of the present program actively targeted sensory management, instruction and counselling, while perceptual training was not explicitly addressed within the program.

The content was initially created by certified clinical audiologists working at the Audiology Clinic, Skåne University Hospital, Lund, Sweden. The content was then fitted on to the platform in close collaboration with staff from the ’Support and Treatment’ design office. Content was created to be presented in chapters. Chapters here refer to a sequential presentation of content.

Four chapters with different content were created. The first introductory chapter aimed to provide information on how the rehabilitation process and the hearing aid intervention work. It also provided material that aimed to prepare the patient to facilitate the needs assessment made face-to-face with the audiologist. The second chapter focused on information about hearing and hearing loss, but also what to expect as a person receiving hearing aid(s). The third chapter focused on how to achieve individual goals with hearing aid use and how to practically manage the specific hearing aids fitted to the individual. The fourth chapter provided information on assistive listening devices and communication strategies. A fifth chapter was added that provided a summary and repetition of previous chapters. Exercises for the individual to do either online or in real life were created for three chapters. An illustrator created graphics to complement the chapter text content and the tasks.

### 2.3 Concurrent review process during content development

To ensure that accessibility, content topics and readability were acceptable for the end-users, four audiologists working at, or in collaboration with the clinic, and seven experienced hearing aid users who had recently received new hearing aid(s) at the clinic reviewed the program at its current state. This was done in parallel with and to inform the finalization of the chapter content as described above. The four certified audiologists with both clinical and research experience reviewed the program and provided feedback on its’ content. Several iterations of revisions were made during content development until a version was ready to be tested with hearing aid users. The seven experienced hearing aid users then tested the login procedures and reviewed the first chapter content based on their experiences as patients and hearing aid users. They provided feedback and suggested adjustments on the present content and what they expected from the following chapters. They also tested the manageability to inform how a login instruction checklist could be compiled. This part of the review process was made in an individual informal think-aloud session together with two audiologists.

In the end, a complete, thoroughly reviewed version of the program was produced. At this stage, since the 1177.se-portal has an accessibility policy regarding content readability, all materials were checked for readability issues.

## 3 Revised program version—outline and content specifications

### 3.1 Outline

The final draft of the online audiological support program consists of four chapters, each with a different focus, and a fifth chapter with repetition of the earlier chapters. Each chapter builds on the previous and relates to the clinical visits. Chapters 2 to 5 start with a review of the previous chapter's content.

The program included a combination of different contents; an initial video welcoming the individual to the program explaining what will happen during the audiological rehabilitation, written texts, exercises, and graphics. The program could be accessed online via smartphones, computers and tablets. The layout of the program and its content are shown in Figure 1 in relation to the clinical visits. Individuals worked with the program in a linear fashion, where access to the next chapter was given once the individual completed the previous chapter and the corresponding clinical visit.

**Figure 1:**
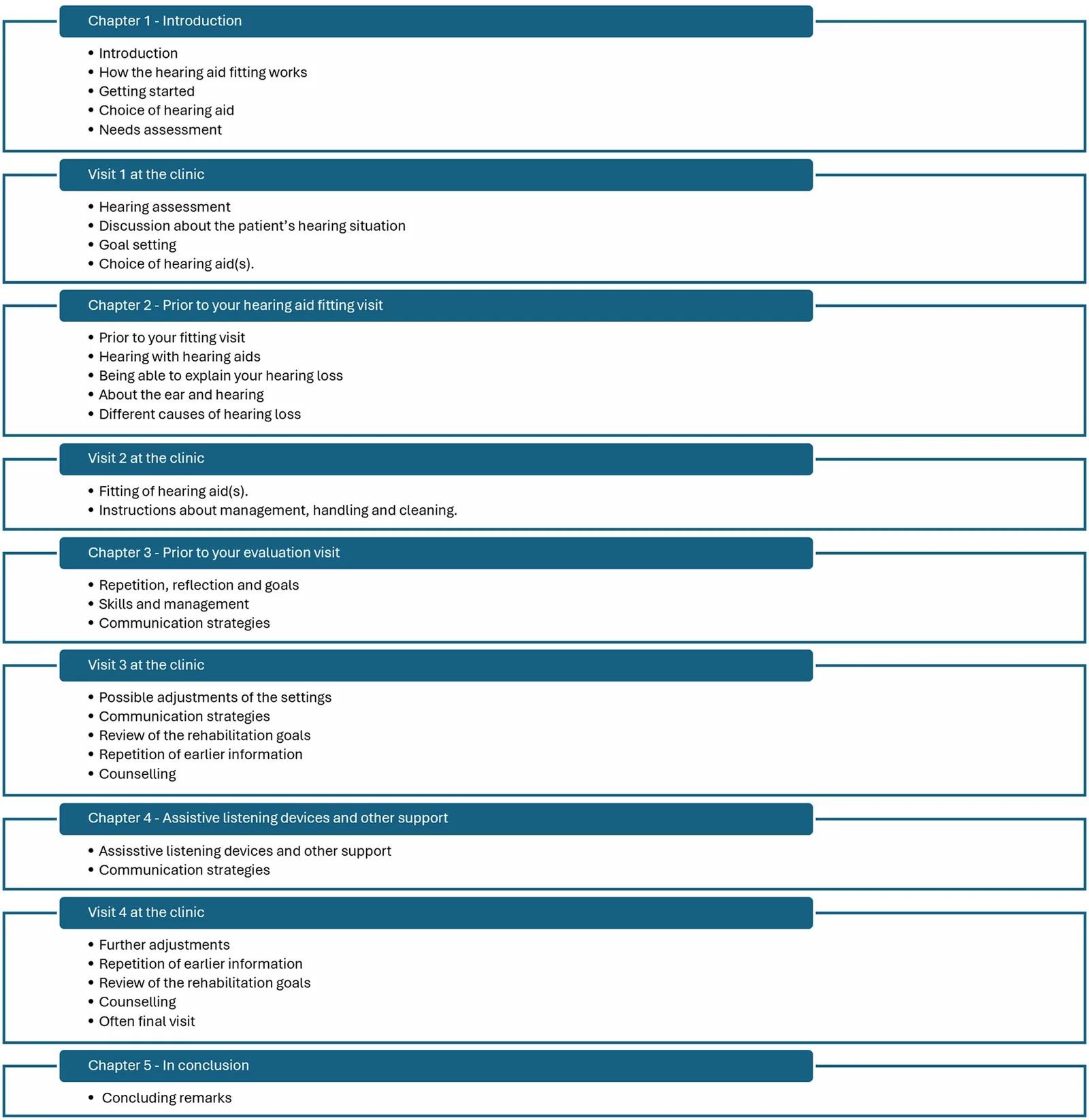
Content of the program chapters in relation to the clinical visits.

### 3.2 Content specifications

The first chapter is intended for use prior to the initial visit to the audiologist. It includes information about what will happen during the upcoming visits at the clinic and how the audiological rehabilitation process works. It introduces the program and informs about motivation, expectations, the ear's prerequisites and the benefits with starting to use hearing aids early. It gives information about the choice of hearing aids and the needs assessment. The individual is asked to think about their situation and their needs so that they can have a more informative discussion with the audiologist at the first clinical visit.

The second chapter is made available to the person after their first visit to the clinic. It includes a task where the individual is asked to evaluate their rehabilitation goals and the chapter gives information about getting used to hearing aids, sound fatigue and tips to adapt to new everyday sounds. It also informs about different hearing aid settings and programs, the compromise between the best possible speech perception and the most comfortable sound, and that the hearing rehabilitation is a collaboration between the patient and the audiologist. They are also encouraged to discuss their situation with the audiologist. Chapter 2 also focuses on why it's important to know about and understand your own hearing loss and being able to explain it to others. The individual learns about how the audiogram is interpreted and they get a task to explain their audiogram to someone else. They also learn about different types of hearing loss and how these can affect hearing.

Chapter 3 is made available to the person following the second visit to the clinic. The third chapter includes information about adjusting to life with hearing aids, communication strategies, an exercise where the person is asked to evaluate their rehabilitation goals this far, and lastly information about handling, management and cleaning of hearing aid(s). The chapter comes in several versions depending on the type of hearing aid fitted. This, to ensure that the individual is provided with relevant, tailored information for his or her type of hearing aid.

Chapter 4 is opened after the third visit and has focus on communication strategies and assistive listening devices like tv-streamers, Bluetooth, telecoil, alerting products and accessories. It also informs about the possibilities of text- and sign language interpretation.

The fifth and final chapter is possible to access after the final visit. It is a brief summary of all the previous chapters and encourages the individual to keep practicing with the hearing aid(s).

## 4 Feedback and insights from first-time and experienced hearing aid users

Thirty-six individuals on the waiting list for receiving hearing aid(s) at the Audiology Clinic, Skåne University Hospital in Lund, Sweden, were invited to test and evaluate the program. Since this version of the program targets adult first-time hearing aid users, participants had to be over 20 years of age, have experience of using the internet and be able to read and speak Swedish. Both first-time and experienced hearing aid users were invited to participate to gather a broad range of feedback on the content and the programs’ manageability and ease of use. The inclusion of experienced hearing aid users was particularly valuable, as their perspective spanning from initial adoption through continued use of hearing aid(s) enabled them to identify needs and challenges that first-time users may not yet recognize.

Thirteen people agreed to participate and to test and evaluate the program during their audiological rehabilitation process to provide feedback and insights. They received hearing aid(s) according to standard clinical praxis and in addition, they tested the program and the process as described in Figure 1. If the participant had not completed a chapter a couple of days prior to their next visit, a reminder was sent via the system chat function. The participants received access to the program via 1177.se using secure e-identification. At physical appointments, they met with one of two clinical audiologists involved in the study (SA or LI).

One person did not have the time and ended their participation before any chapter had been provided. One person stopped after the provision of the second chapter without stating their reason for quitting. Eight participants completed and reviewed all five chapters while three completed all except the last repetition chapter. The group consisted of five men and six women (mean age 55.5 years, range 34-76). There were four first-time hearing aid users and seven experienced users with prior hearing aid experience ranging from two months to over 20 years.

### 4.1 Materials used for structured feedback

The participants shared their experiences and opinions related to the comprehension and relevance of the content by answering 7 questions in relation to each chapter and by answering five questions about manageability and ease of use. After completion of the program, they also evaluated 16 statements about their general experience of the program. The use of structured questions was deemed a pragmatic way to gather information, and in addition, free-text fields provided participants with the opportunity to elaborate on their responses, offering deeper insight and more detailed suggestions for improvement and change. Free-text answers were not subject to formal qualitative analysis and are presented only as representative examples of the written comments received from participants.

First, at the conclusion of each chapter, the participants answered three questions online: 1. *How did you experience the chapter?* (response options; “Very poor”, “Poor”, “Neither”, “Good” or “Very good”), 2. *Was the content of the chapter useful to you?* (response options; “Not useful”, “Neither”, “Useful” or “Very useful”) and 3. *Approximately, how long did it take to finish the chapter?* (response options; “<30 min”, “30-60 min” or “>60 min”). They also responded to four yes/no questions on paper with similar content as in Werther, Thorén (32). These questions targeted content information accessibility and content relevance. They handed in their responses at the next clinical appointment. The questions were: 1. *Did you find the information in the chapter clear?*, 2. *Did you find any of the information or text in the chapter too difficult?*, 3. *Did you find any of the information in the chapter irrelevant?* and 4. *Did you find that any information in the chapter was missing?*.

After the completion of all chapters in the program, the participants answered five yes/no questions about program manageability and ease of use: 1. *Did you ever have problems with logging in to the program?*, 2. *Did you ever encounter any technical difficulties when using the program?*, 3. *Did you ever experience that it was difficult to navigate in the program?*, 4. *Would you rather have received the information in paper form than online?* and 5. *Did the program give you any information that you benefit from in your day-to-day living?.* If they responded yes to any of these questions, they were asked to explain in writing in which way.

They also rated to what extent they agreed with each of 16 statements regarding the general experience of the program, with focus on comprehension and relevance of the content and, manageability and ease of use. Inspiration for these items came from Ferguson, Maidment (28). The response options for each item were “Agree”, “Neither agree nor disagree” and “Disagree”. The participants also answered additional questions not reported on here.

### 4.2 Review outcomes and feedback collected during ongoing program

The review thus resulted in responses to questions and written feedback. These outcomes are presented descriptively. Most participants answered all questions, however, some forgot to submit their responses.

The frequency of the responses collected directly after the completion of each chapter is shown in Table 1. All participants found the content of the chapters good or very good and most found it useful. Chapter 4, which focuses on assistive listening devices, communication strategies and information about sign language- and text interpretation, was deemed less useful than the other chapters. It took no more than 60 min to complete each chapter.

**Table 1:** Frequency distribution of online responses directly after the completion of each chapter.

Question	Response	Chapter				
		1 (*n* = 11)	2 (*n* = 11)	3 (*n* = 11)	4 (*n* = 11)	5 (*n* = 8)
How did you experience the chapter?	Very poor	-	-	-	-	-
	Poor	-	-	-	-	-
	Neither	-	-	-	-	1
	Good	8	7	6	11	5
	Very good	3	4	5	-	2
Was the content of the chapter useful to you?	Not useful	-	-	-	-	-
	Neither	3	-	1	6	2
	Useful	7	8	7	5	6
	Very useful	1	3	3	-	-
Approximately how long did it take to finish the chapter?	<30 min	7	7	8	10	8
	30–60 min	4	4	3	1	-
	>60 min	-	-	-	-	-

Furthermore, the participants answered five yes-/no questions regarding content information accessibility and content relevance. As seen in Table 2, the answers suggested that the information and content were regarded as clear by all and mostly relevant. Chapter 4 was again reported to be less relevant than the other chapters.

**Table 2:** Participant feedback given via paper form after completion of each chapter.

Question	Response	Chapter				
		1	2	3	4	5
Did you find the information in the chapter clear?	Yes No	11 (100%) -	10 (100%) -	8 (100%) -	8 (100%) -	10 (100%) -
Did you find any of the information or text in the chapter too difficult?	Yes No	- 11 (100%)	2 (20%) 8 (80%)	- 8 (100%)	1 (12,5%) 7 (87,5%)	- 10 (100%)
Did you find any of the information in the chapter irrelevant?	Yes No	- 11 (100%)	1 (10%) 9 (90%)	- 8 (100%)	2 (40%) 6 (60%)	1 (10%) 9 (90%)
Did you find that any information in the chapter was missing?	Yes No	2 (18%) 9 (82%)	2 (22%) 7 (78%)	2 (25%) 6 (75%)	1 (12,5%) 7 (87,5%)	- 10 (100%)

Chapter 1: *n* = 11, chapter 2: *n* = 10 (except for the last question, *n* = 9), chapter 3: *n* = 8, chapter 4: *n* = 8, chapter 5: *n* = 10.

Two free-text comments regarding the difficulty of the chapter content were obtained. One participant commented that it would have been valuable with even more information regarding the anatomy of the ear and the difference between conductive and sensorineural hearing loss in chapter 2, as they experienced that the content raised more questions about different types of hearing loss. Another participant provided a grammatical observation in chapter 4, which could make the information clearer.

A few comments on missing information or requests for more information were also provided by the participants. These comments were about tinnitus (two participants), features of different hearing aids including telecoils and the localization of sounds (one participant) and more in-depth information on auditory physiology and the possibility to see notes from earlier visits in the clinic (one participant).

### 4.3 Review outcomes collected after the completion of the program

To understand whether the ’Support and Treatment’ platform was suitable for the delivery of the program, the participants answered five questions related to the program manageability and ease of use, see Table 3. These questions showed that some participants had problems finding the program at the national health portal and thought it was difficult to log in and navigate the first time. All but one reported that the program gave them information that was beneficial in their daily lives. They were also asked if they would prefer to receive the same information as given through the program but in paper format. A few participants stated that they would have wanted both, but no one preferred paper format exclusively.

**Table 3:** Participant responses and feedback regarding manageability and ease of use, answered in paper form after the completion of the last chapter.

Question	Response	Frequency (%)	Free-text answers linked to the question
Did you ever have problems with logging in to the program? If yes, please explain in which way.	Yes No	3 (30%) 7 (70%)	*“It was difficult to find the program at 1177” (P13)* *“In the beginning, before I understood how it worked” (P19)* *“The first time before I understood the structure of it” (P27)*
Did you ever encounter any technical difficulties when using the program? If yes, please explain in which way.	Yes No	4 (40%) 6 (60%)	*“It was difficult to find the program at 1177” (P13)* *“At some point 1177 acted up” (P21)* *“The message function was maybe a little slow sometimes” (P22)* *“Yes, it said that there was a new chapter, but when I logged on there was no new chapter” (P25)*
Did you ever experience that it was difficult to navigate in the program? If yes, please explain in which way.	Yes No	2 (25%) 6 (75%)	*“Except for finding the program at 1177—no” (P13)* *“In the beginning, but I looked around and quite fast got the hang of it!” (P19)*
Would you rather have received the information in paper form than online? If yes, please explain why.	Yes No	2^a^ 9 (100%)	*“No, but it would have been good to get the communication strategies printed” (P13)* *“Both yes and no, for those who aren't used to computers or as me who thinks it's easier to go back in paper than on the computer” (P25)* *“Yes, preferably both, especially for older first-time users since they are more used to written information” (P27)*
Did the program give you any information that you benefit from in your day-to-day living? Please explain how.	Yes No	9 (90%) 1 (10%)	*“The program probably made me feel more confident in myself, and therefore I now use the hearing aid with greater confidence!” (P3)* *“Strategies for use and instructions related to the hearing aid like cleaning for example” (P13)* *“Absolutely! I got recognition regarding my problems. And many of the problems are today solved” (P19)* *“The advice for me and my close ones were good” (P21)* *“Good info. Some things I would have liked to get in paper form. For example, what [sic] and when I should not wear the hearing aid. I’m a bit unsure now when I’m going to a concert. I think I should take it off. This I would have liked to have in paper form. And how to clean the hearing aid” (P25)* *“No, since I’ve had hearing aids for some years” (P33)*

Please note that not all participants answered all questions. ^a^These two participants wanted both.

Table 4 shows the ratings for the 16 items regarding the general experience with the program. The participants stated that the program would be useful for first-time hearing aid users, and all would recommend it to someone else. Most of the participants felt motivated to use the program, but few stated that they wanted to actively use it again after the audiological rehabilitation was completed. They appreciated the content order in the program, and all participants followed the intended program structure. Most felt that the program increased their confidence regarding their own hearing and hearing aid(s) and that the program encouraged them to use their hearing aid(s). None of the participants had actively showed the program to a communication partner. The usability was deemed good by all but one participant. No one stated that the workload had been too heavy.

**Table 4:** Ratings for 16 items regarding the general experience of the program answered in paper form after the completion of the last chapter.

Statement	Agree	Neither agree nor disagree	Disagree
I found the information in the program relevant^a^	9 (90%)	1 (10%)	-
I think that someone who gets their first hearing aid would benefit from the program	11 (100%)	1^b^	-
I would like to use the program again	3 (27%)	8 (73%)	-
I felt motivated to use the program	9 (90%)	-	1 (10%)
The content of the program was interesting	10 (91%)	1 (9%)	-
I found that the distribution between text and pictures was good	10 (91%)	1 (9%)	-
I thought the order of the chapters was good	10 (91%)	1 (9%)	-
I did not follow the program structure, but jumped between the sections which seemed most interesting	-	-	11 (100%)
I looked through all the material	10 (91%)	1 (9%)	-
I feel that the program has helped me to become more confident about my hearing and my hearing aid(s)	7 (64%)	4 (36%)	-
The program encouraged me to use my hearing aid(s)	7 (64%)	3 (27%)	1 (9%)
I showed the program to a communication partner	-	3 (27%)	8 (73%)
The program gave me information that I did not get from my audiologist	3 (27%)	6 (55%)	2 (18%)
I would recommend the program to someone else	11 (100%)	-	-
I felt that the program had good usability	10 (91%)	1 (9%)	-
I felt that the workload of the program was OK	10 (91%)	1 (9%)	-

a One participant did not answer this question, ^b^One participant marked both answers.

Furthermore, free-text comments were collected after each chapter and at the completion of the program. The participants were generally satisfied with the content and progress of their audiological rehabilitation. Comments were made on individual chapters. One participant stated that chapter 1, aiming to prepare the individual for the upcoming rehabilitation process and the needs assessment, “*Gave me answers to questions I had and stuff I had not thought about, but which are relevant to know” (P13).* In connection to chapter 2, with focus on preparing the individual for the fitting visit and providing information on the ear's anatomy and understanding of one's hearing loss, another participant specified that “*Everything feels relevant and the process safe and thorough” (P19)*. Regarding chapter 3, which focuses on the first impressions of using hearing aids and self-management regarding hearing aids, one participant wrote *“Great to get another review of the hearing aid cleaning. I needed that since the visits include a lot of information and I felt a bit unsure about the cleaning of the hearing aid. Also good to be able to reflect upon my goals and write about how I feel, what I think, and what I am experiencing. I would have liked to have easier access to my goals which emerged at my first visit” (P13).* Another participant stated, “*Well, very clear and good. Very useful info. Great. Nice pictures that clarify if you have difficulties with the Swedish language, then you can understand by looking at the pictures” (P25)*.

Several comments were also obtained from the experienced hearing aid users regarding the content not being new or relevant to them specifically, but more aligned with the needs of first-time hearing aid users: “*Since I’ve had hearing aids for some years the content is familiar” (P27), “(…) addressed to new hearing aid users” (P22)* and “*Sometimes a little too basic and obvious for those who are not first-time users” (P21)*.

The information provided through the program was regarded as clear by all, and one participant commented, in connection to chapter 1, that it was “*Informative and with supportive pictures if you would be unsure” (P25).* But, as seen in Tables 1 and 2, chapter 4 was regarded as less useful and relevant for them, one participant stated: “*A lot of the information was targeted at people with a greater hearing loss than I have” (P21)* and another commented “*Good with information about possibilities that might be needed later on” (P11)*.

## 5 Lessons learned

An online support program for the audiological rehabilitation process aimed at adult first-time hearing aid users was developed based on previous studies on audiological rehabilitation, clinical expertise and with hearing aid users’ insights. The program was reviewed and tested in several iterations with feedback from certified clinical audiologists, hearing aid users and staff on the ’Support and Treatment’ platform to refine its content and manageability. Experiences with the program were examined through participant feedback on the comprehension and relevance of the content and the manageability and ease of use of the program. Figure 2 shows the development process from content development to the final version of the program. Overall, participants provided positive feedback on the program. The feedback provided by the participants described the program content as clear, useful and relevant, but with comments that the program mainly targeted first-time hearing aid users’ needs. This suggests that the program targets the needs of the intended patient group, first-time users. The feedback from the experienced hearing aid users indicated that the program did not miss any relevant information that could be useful for first-time hearing aid users. Chapter 4 provided information on assistive listening devices and was regarded as less useful than the others. It is possible that the small study sample did not represent patients that are eligible for or would benefit from assistive listening devices.

**Figure 2:**
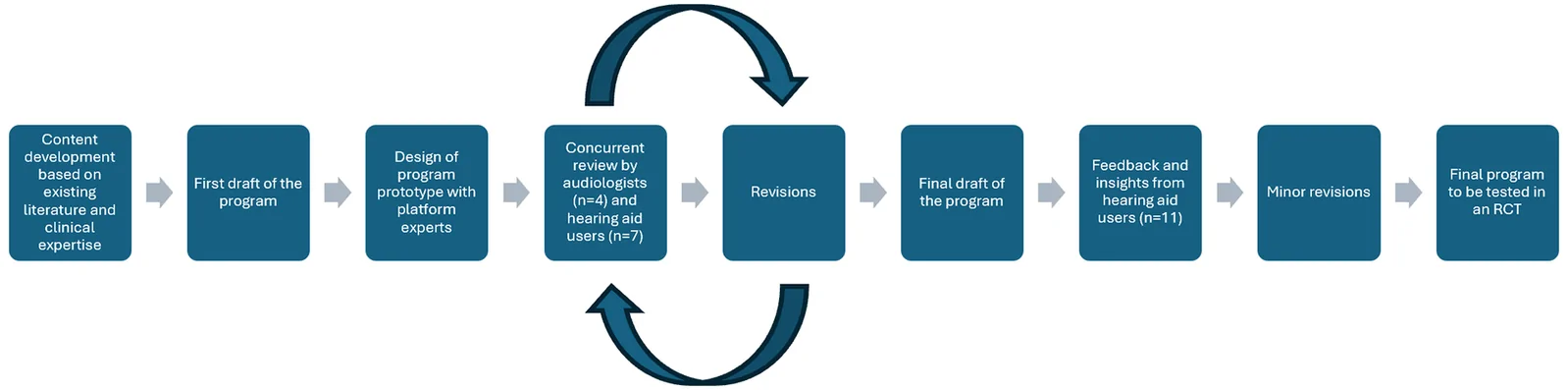
Development process.

Based on the outcomes of the final review and evaluation process some minor changes were made to the program. Some of the pictures were changed since these included the old version of the Bluetooth symbol. Other pictures were updated to clearly explain the audiogram and new information about rechargeable hearing aids were added.

The time it took to complete the chapters, and the workload of the program was deemed to be acceptable, not exceeding 60 min. The program was reported to be manageable and easy to access and use, even if some participants had trouble logging in to the program at the national health portal (1177.se) the first time. This finding is in line with other studies that have used the same platform (31, 32). Therefore, it was deemed important to update the login instruction checklist for users to improve future access to the platform. Once logged on to the national health portal, it was easy to navigate the program and participants reported good usability.

Remote interventions for people with hearing problems have been proven to be able to provide easy access to personalized information in an interactive and engaging way (36). The program in the present study is the first to use a combination of structured chapters used in parallel with face-to-face visits at the clinic to guide and support individuals receiving audiological rehabilitation with hearing aids. As individuals can prepare prior to their clinical visit, appointments can then focus on more in-depth discussions and personalized adjustments rather than repeating information. Further, for the audiologist the program facilitates the possibility to provide multiple individuals with support simultaneously and provide personalized feedback when needed. Since it was mandatory for the participants to complete the different chapters of the program, the program now developed differs from other, more or less similar ones, as the support system by Brännström et al. (37) that provided support via a resource library where the audiologist made information available based on individual needs with a recommendation to the patient to use this resource. The present program is also different from C2Hear and m2Hear which are two multimedia educational programs designed to complement the clinical rehabilitation practices by providing individualized learning opportunities where the user was free to view what they deemed relevant based on individual needs (28, 29).

The sample in the present study is representative of the patients at Audiology Clinic, Skåne University Hospital, Lund, Sweden, but not for all people with hearing problems. This clinic rarely sees patients with age-related hearing loss, since patients at this clinic usually have a hearing problem that requires some kind of medical treatment. It is possible that a more diverse group of people with different kinds of hearing loss, age, digital literacy, and internet experience could have provided more information on, for example, missing content. The small sample size, together with the demographics of the participants, limits the applicability of the results. A future study investigating the needs of a more diverse group of participants would strengthen the understanding of the usefulness of the program and if other ways of delivering such support is warranted. Further, an interview study or focus group study with both people with hearing loss and/or hearing aids, and clinical audiologists could deepen the understanding of the program and its usability.

The review and evaluation process was conducted using a number of questions. Though the questions were inspired by other studies, validated questionnaires should preferably be used. The System Usability Scale is a questionnaire that assesses management and ease-of-use (38). However, a validated Swedish version was not available at the time of the study (39). In hindsight, it would have been valuable to use questions about the reflection tasks in the program and if the participants wanted more video content. Video content is used to a high degree in similar educational programs, like C2Hear, m2Hear and SUPR which have been shown to be effective (14, 19, 28). Although the participants were given several opportunities to relay information on missing parts of the program, topic prompts could be used to remind participants of things to consider in their review and feedback.

Information on individual usage time could not be collected due to platform limitations. In future studies, detailed user log files would improve further understanding of what information was most and least useful to the hearing aid users.

## 6 Conclusions

An online support program for the audiological rehabilitation journey was developed to be used in parallel with face-to-face visits at the clinic. Both first-time hearing aid users and experienced users who evaluated and tested the program found it relevant, manageable and easy to use. However, the content was mostly regarded to be aligned with first-time hearing aid users’ needs, and the chapter with content about assistive listening devices was deemed less relevant. Further, some participants reported technical difficulties regarding logging in to the Swedish national health portal. The possible effects of the program will be tested in an RCT.

## Data Availability

The datasets presented in this article are not readily available because all data are handled according to the Swedish Act concerning the ethical review of research involving humans to attain confidentiality. Requests to access the datasets should be directed to sandra.ahlberg@med.lu.se.
